# Supplementary material for: The Drosophila drop-dead gene is required for eggshell integrity
Source: PLoS One. 2023 Dec 5;18(12):e0295412. doi: 10.1371/journal.pone.0295412 (PMC10697589; doi:10.1371/journal.pone.0295412)
Supplement: S1 Fig — (A) Genomic DNA sequences. Upper sequence shows the wild-type sequence of the final five bases of exon 8 (bold), all of intron 8, and the first five bases of exon 9 (bold). The lower sequence shows the same region in drd1, with the single base change (green, underlined) and the new start of exon 9 (bold). (B) cDNA sequences. Upper sequence shows the wild-type sequence of cDNA from the region of the exon 8/9 junction. The splicing site is indicated in red. The lower sequence shows the same region in drd1, with the new splice junction shown in red and the added 10 bases in bold. (PDF) [file pone.0295412.s003.pdf]

A: genomic DNA

wild-type

TAATTGTAGGTATTCGCCCAGGTCTAGCATTTATAGAGAATTGTATTTTTTGTTGTTTTTTATTTGCTCAACGCAG**CTCGA**

drd^{1}

TAATTGTAGGTATTCGCCCAGGTCTAGCATTTATAGAGAATTGTATTTTTTGTTGTTTTTTATTAG**CTCAACGCAGCTCGA**

B: cDNA

wild-type CAACTTTGTTTCGATAATTCTCGATACCAACGAATTCATATCG

drd^{1} CAACTTTGTTTCGATAATT**CTCAACGCAG**CTCGATACCAACGAATTCATATCG
