# Supplementary figures and images for: The Drosophila drop-dead gene is required for eggshell integrity
Source: PLoS One. 2023 Dec 5;18(12):e0295412. doi: 10.1371/journal.pone.0295412 (PMC10697589; doi:10.1371/journal.pone.0295412)

A

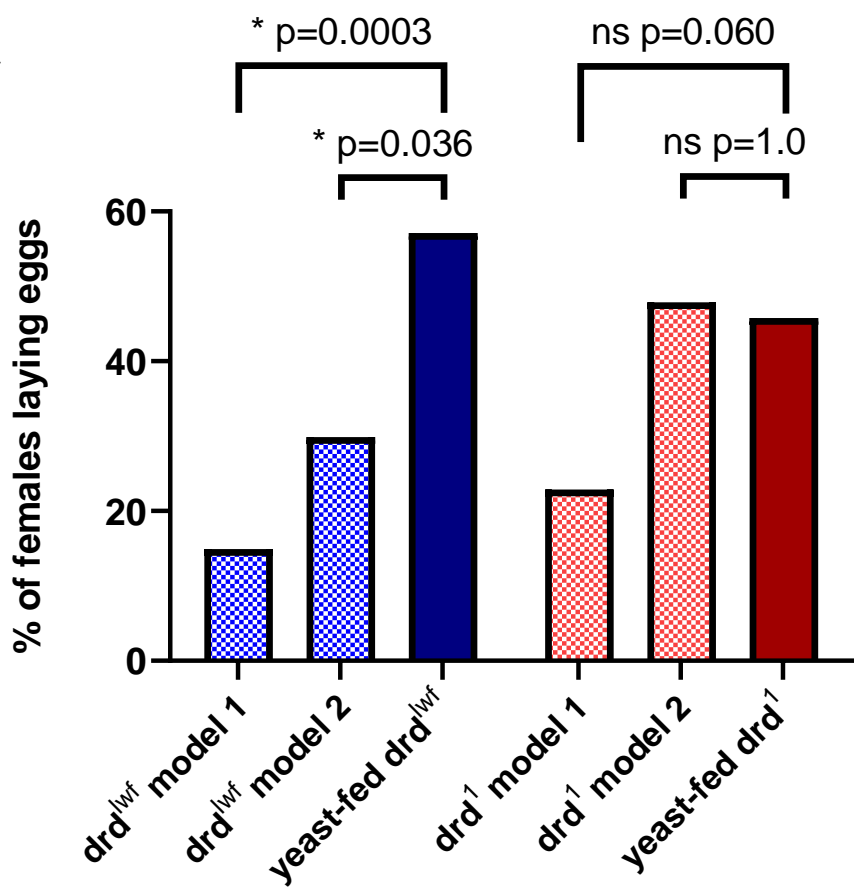

B

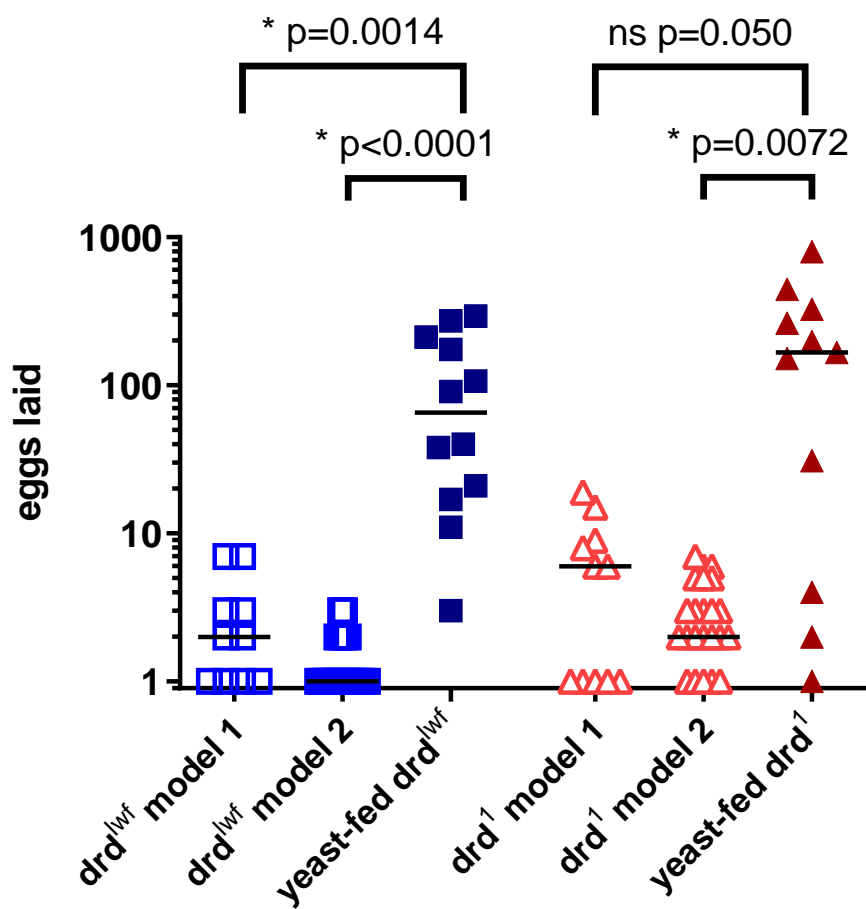

Supplement: S2 Fig — (A) Fraction of females in each condition that laid any eggs during their lifetime. Because some non yeast-fed females were assayed in groups of 2–3, the data were analyzed with two different assumptions regarding the distribution of egg-laying (see methods). Brackets indicate the effect of yeast feeding (two-sided Fisher’s exact test). (B) Number of eggs laid per female, omitting data from those females that laid no eggs. Brackets indicate the effect of yeast feeding (Kruskal-Wallis test with Dunn’s post-hoc multiple comparisons test). Blue: drdlwf females; Red: drd1 females. n = 67 drdlwf females, 21 yeast-fed drdlwf, 48 drd1, 24 yeast-fed drd1. (PDF) [file pone.0295412.s004.pdf]

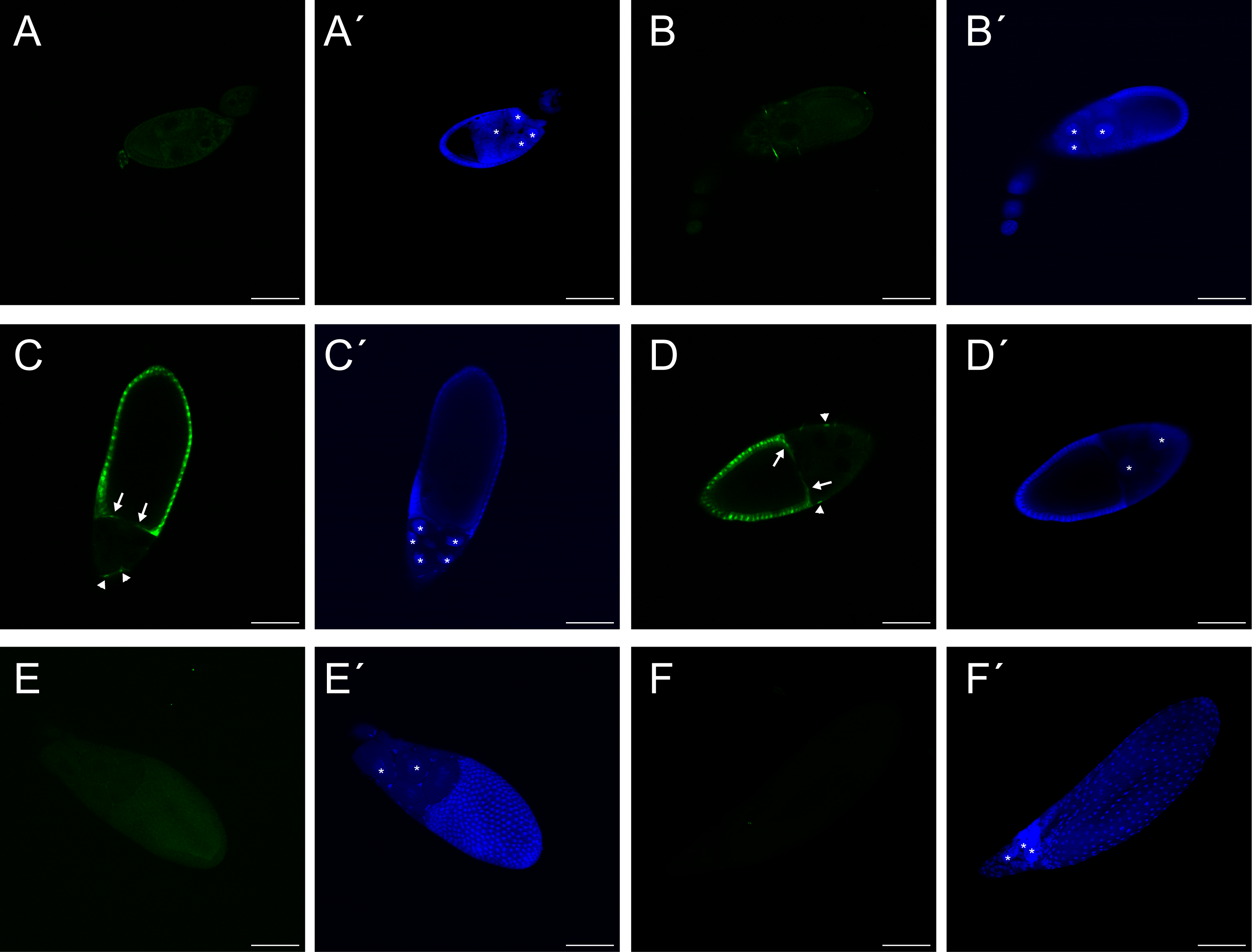

Supplement: S3 Fig — (A-F) GFP expression driven by the drd-GAL4 driver. (A´-F´) DAPI staining of cell nuclei, with nurse cell nuclei in the anterior of each egg chamber indicated with asterisks. (A, A´) A stage 9 egg chamber, showing no GFP labeling. (B, B´) A stage 10A egg chamber, showing no GFP labeling. (C, C´) A stage 11 egg chamber, showing labeled follicle cells. (D, D´) A stage 10B egg chamber showing labeled follicle cells. Labeled stretch and centripetal follicle cells in C and D are indicated with arrowheads and arrows, respectively. (E, E´, F, F´) Control stage 10B (E, E´) and stage 12 (F, F´) egg chambers lacking the drd-GAL4 transgene. A-D are single confocal images, and E-F are maximum intensity projections of Z-stacks. Scale bar is 100 μm. (TIF) [file pone.0295412.s005.tif]

A

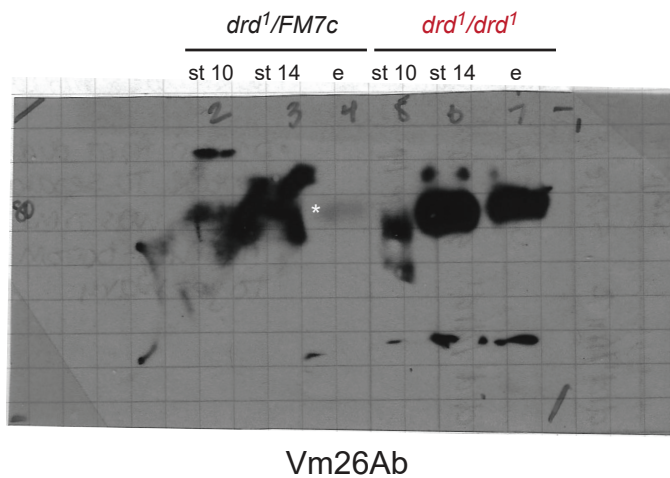

B

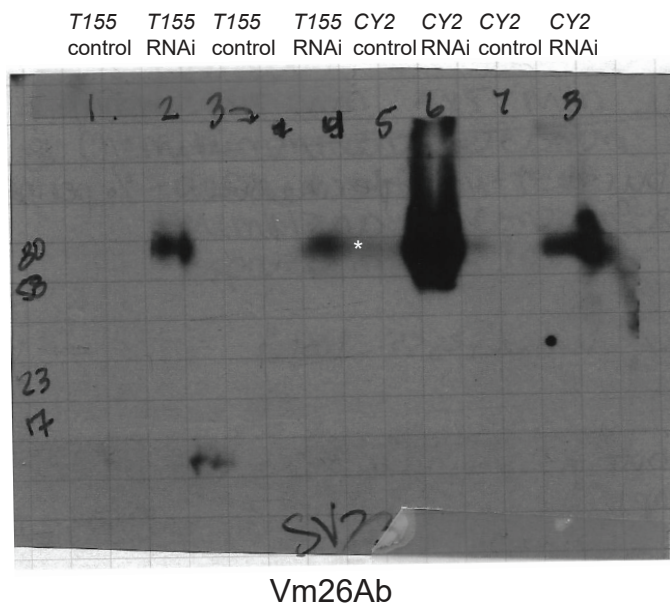

C

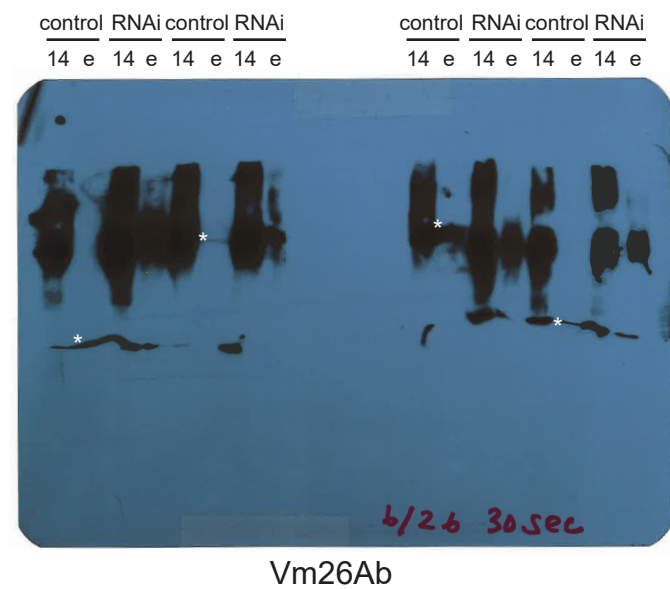

Supplement: S4 Fig — (A) Stage 10 and 14 egg chambers dissected from drd1/FM7c heterozygous and drd1/drd1 mutant females, as well as eggs (e) laid by these females that were collected 0–5 hr after oviposition. 2 eggs or egg chambers per lane (except mutant st 10, 1 egg chamber), 1:25,000 primary antibody dilution. (B) Laid eggs collected 0–4.5 hr after oviposition. Lane 1: w; CyO/+; T155-GAL4/+, (sibling controls of lane 2); Lane 2: w; UAS-Dcr-2 drdGD3367/+; T155-GAL4/+; Lane 3: w; CyO/+; T155-GAL4/+ (sibling controls of lane 4); Lane 4: w; drdGD15915 UAS-Dcr-2/+; T155-GAL4/+; Lane 5: w; CY2-GAL4/CyO (sibling controls of lane 6); Lane 6: w; UAS-Dcr-2 drdGD3367/CY2-GAL4; Lane 7: w; CY2-GAL4/CyO (sibling controls of lane 8); Lane 8: w; drdGD15915 UAS-Dcr-2/CY2-GAL4. 5 eggs per lane, 1:10,000 primary antibody dilution. (C) Stage 14 egg chambers and laid eggs collected 0–3.5 h after oviposition. Lanes 1–2: w; CY2-GAL4/CyO (sibling control of lanes 3–4); Lanes 3–4: w; drdGD15915 UAS-Dcr-2/CY2-GAL4; Lanes 5–6: w; CY2-GAL4/CyO (sibling control of lanes 7–8); Lanes 7–8: w; UAS-Dcr-2 drdGD3367/CY2-GAL4. Left gel, 2 eggs or egg chambers per lane; right gel, 1 egg or egg chamber per lane, 1:25,000 primary antibody dilution. Asterisks indicate signals in lanes containing laid eggs from control females. (PDF) [file pone.0295412.s006.pdf]

**A**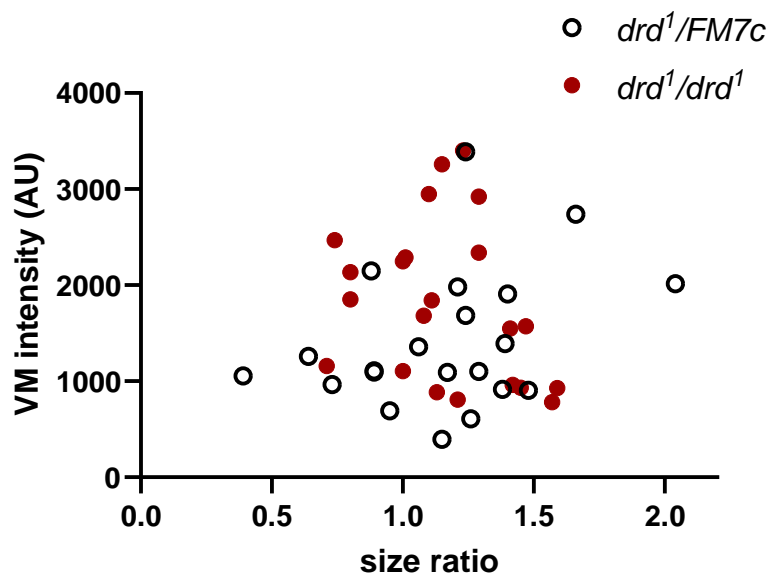**B**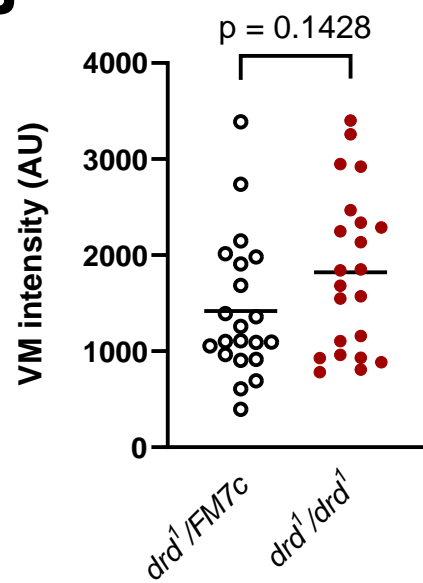**C**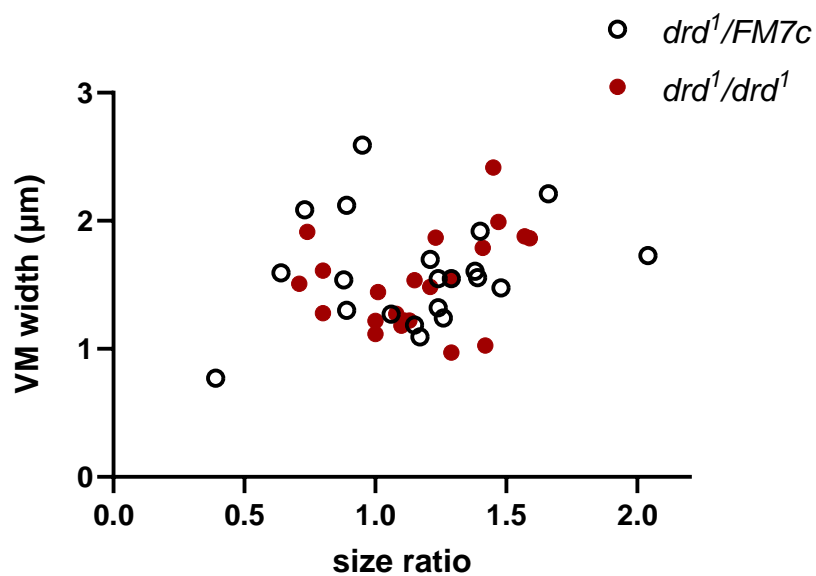**D**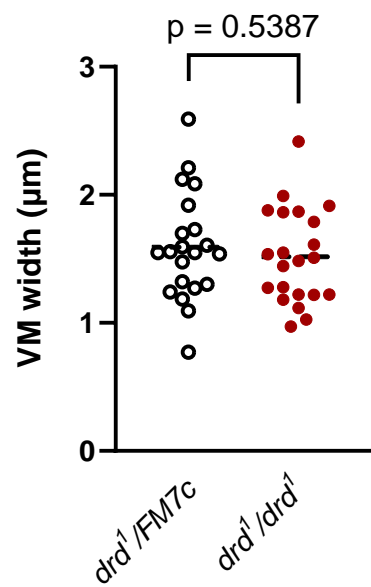

Supplement: S5 Fig — Black open circles: heterozygous controls; red closed circles: drd1 homozygotes. (A) Plot of VM intensity measurements as a function of oocyte size ratio (A-P length/lateral width). There is no significant linear correlation between VM intensity and size ratio for either heterozygotes (p = 0.13) or homozygotes (p = 0.19, F test). (B) Comparison of VM intensity measurements between heterozygotes and homozygotes. Horizontal bars represent the means. The p-value is from a Mann-Whitney test comparing the two genotypes. (C) Plot of VM width measurements as a function of oocyte size ratio (A-P length/lateral width). There is no significant linear correlation between VM width and size ratio for either heterozygotes (p = 0.40) or homozygotes (p = 0.13, F test). (D) Comparison of VM width measurements between heterozygotes and homozygotes. Horizontal bars represent the means. The p-value is from a t-test comparing the two genotypes. n = 21 heterozygotes and 22 homozygotes. (PDF) [file pone.0295412.s007.pdf]
