# Supplementary material for: The Drosophila drop-dead gene is required for eggshell integrity
Source: PLoS One. 2023 Dec 5;18(12):e0295412. doi: 10.1371/journal.pone.0295412 (PMC10697589; doi:10.1371/journal.pone.0295412)
Supplement: S1 Raw images — (PDF) [file pone.0295412.s008.pdf]

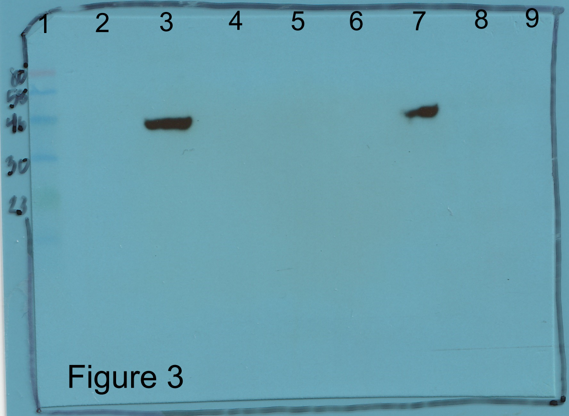

Figure 3

Blot for Figure 3.

Lane 1: MW markers

Lane 2: *drd*^{1}*/drd*^{1}, stage 10 egg chamber

Lane 3: *drd*^{1}*/drd*^{1}, stage 14 egg chamber

Lane 4: *drd*^{1}*/drd*^{1}, laid eggs 0-3 hours after oviposition

Lane 5: *drd*^{1}*/drd*^{1}, laid eggs 0-6 hours after oviposition

Lane 6: *drd*^{1}*/FM7c*, stage 10 egg chamber

Lane 7: *drd*^{1}*/FM7c*, stage 14 egg chamber

Lane 8: *drd*^{1}*/FM7c*, laid eggs 0-3 hours after oviposition

Lane 9: *drd*^{1}*/FM7c*, laid eggs 0-6 hours after oviposition

4 egg chambers or eggs per lane. Anti-Cp36, 1:5000.

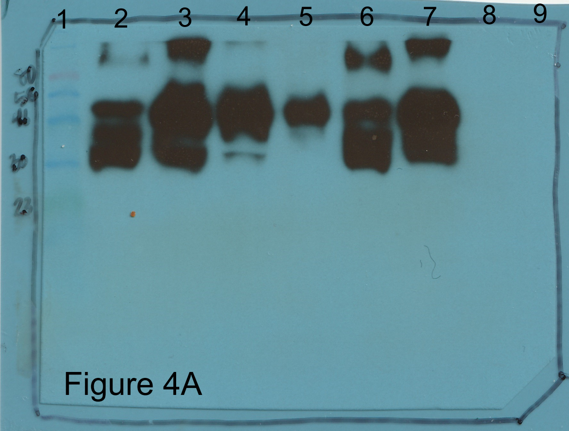

Figure 4A

Blot for Figure 4A

Lane 1: MW markers

Lane 2: *drd*^{1}/*drd*^{1}, stage 10 egg chamber

Lane 3: *drd*^{1}/*drd*^{1}, stage 14 egg chamber

Lane 4: *drd*^{1}/*drd*^{1}, laid eggs 0-3 hours after oviposition

Lane 5: *drd*^{1}/*drd*^{1}, laid eggs 0-6 hours after oviposition

Lane 6: *drd*^{1}/*FM7c*, stage 10 egg chamber

Lane 7: *drd*^{1}/*FM7c*, stage 14 egg chamber

Lane 8: *drd*^{1}/*FM7c*, laid eggs 0-3 hours after oviposition

Lane 9: *drd*^{1}/*FM7c*, laid eggs 0-6 hours after oviposition

4 egg chambers or eggs per lane. Anti- Vm26Ab, 1:10,000.

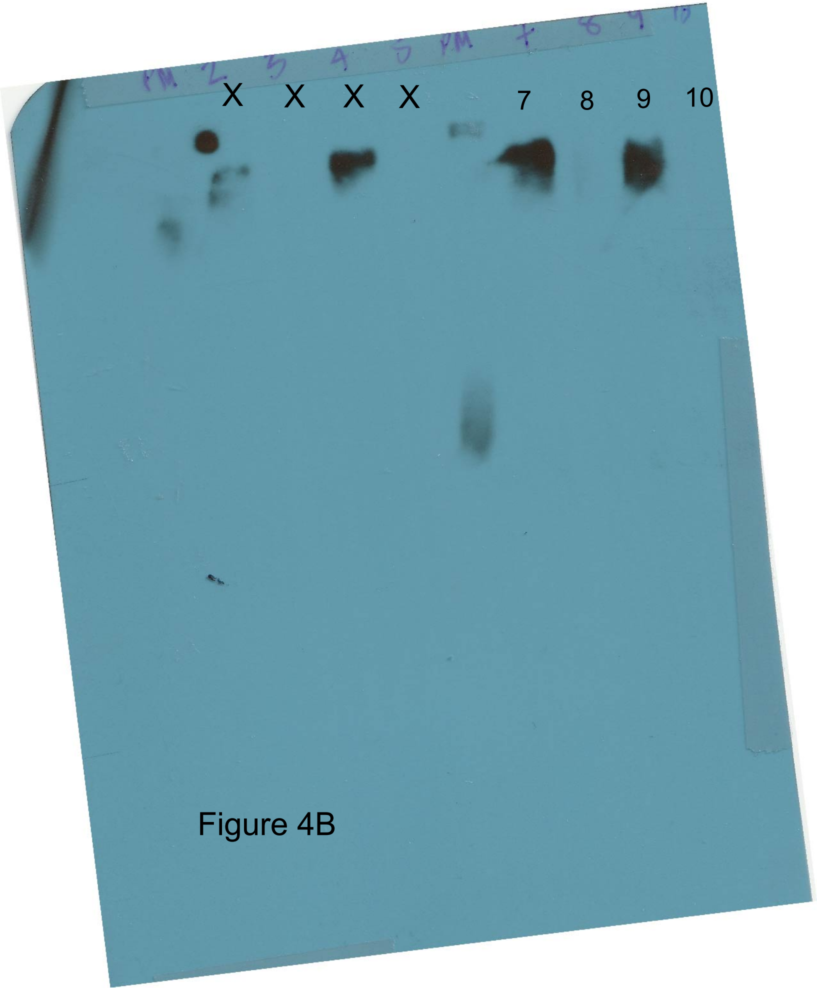

Figure 4B

Blot for Figure 4B

Lane 7: *drd*^{1}*/FM7c*, stage 10 egg chamber

Lane 8: *drd*^{1}*/FM7c*, stage 14 egg chamber

Lane 9: *drd*^{1}*/drd*^{1}, stage 10 egg chamber

Lane 10: *drd*^{1}*/drd*^{1}, stage 14 egg chamber

2 egg chambers per lane, no reducing agents added. Anti- Vm26Ab, 1:10,000.

Note that lanes 2-5 are the same samples, same antibody, 1:25,000 dilution.

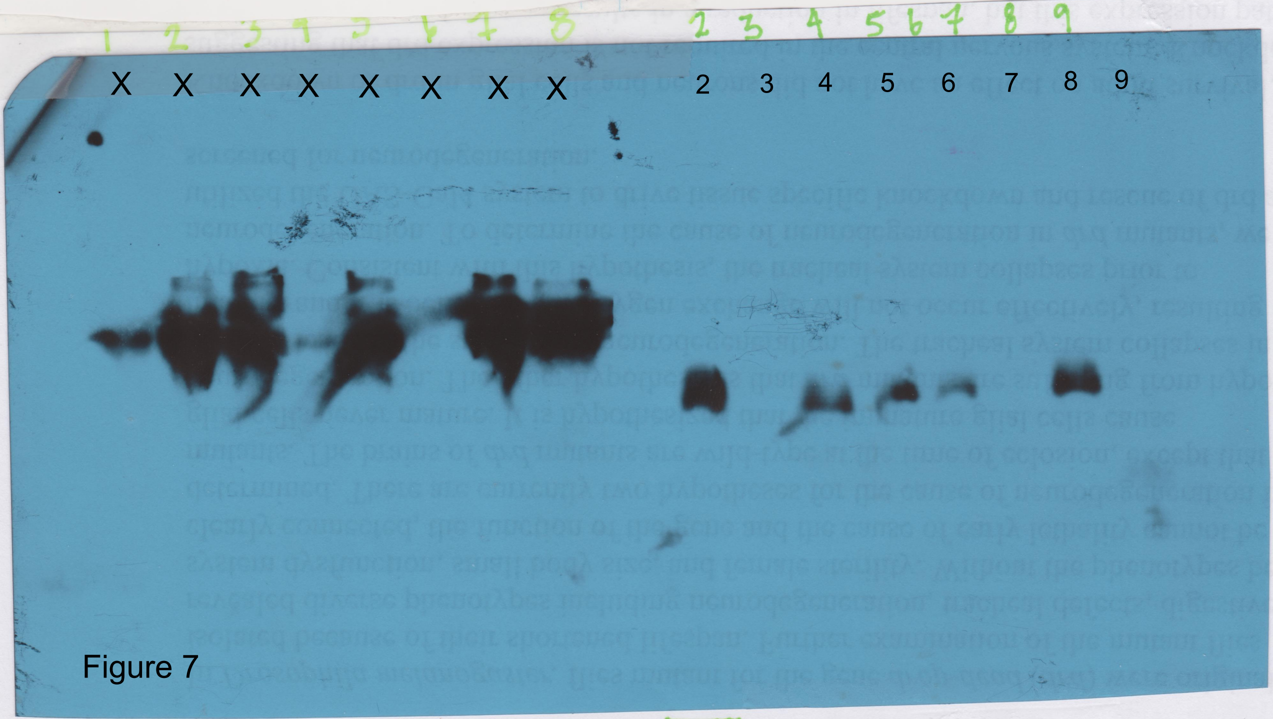

Figure 7

Blot for Figure 7

Lane 2: *w; CY2-GAL4/CyO*, stage 14 egg chambers

Lane 3: *w; CY2-GAL4/CyO*, laid eggs

Lane 4: *w; UAS-Dcr-2 drd*^{GD3367}*/CY2-GAL4*, stage 14 egg chambers

Lane 5: *w; UAS-Dcr-2 drd*^{GD3367}*/CY2-GAL4*, laid eggs

Lane 6: *w; CyO/+; T155-GAL4/+*, stage 14 egg chambers

Lane 7: *w; CyO/+; T155-GAL4/+*, laid eggs

Lane 8: *w; UAS-Dcr-2 drd*^{GD3367}*/+; T155-GAL4/+*, stage 14 egg chambers

Lane 9: *w; UAS-Dcr-2 drd*^{GD3367}*/+; T155-GAL4/+*, laid eggs

2 egg chambers or eggs per lane. Anti- Vm26Ab, 1:25,000.
