# Supplementary material for: The Drosophila drop-dead gene is required for eggshell integrity
Source: PLoS One. 2023 Dec 5;18(12):e0295412. doi: 10.1371/journal.pone.0295412 (PMC10697589; doi:10.1371/journal.pone.0295412)
Supplement: Attachment — Submitted filename: Sheahan et al PLoS One rev1 response to reviewers.docx [file pone.0295412.s009.docx]

**Sheahan et al., response to reviewers**

We thank the reviewers for their close reading of the manuscript and thoughtful comments. Our response to each comment is shown below in blue.

Reviewer #1: This study, “Drosophila drop-dead gene is required for eggshell integrity” by Sheahan and colleagues, investigates the previously observed reduction in egg-laying of drop-dead (drd) mutant females. This phenotype was previously hypothesized to be due to starvation of drd mutant females because of defects in the gut epithelium. Upon feeding with a more nutritious diet (wet yeast), the drd females laid more eggs, but the researchers observed defects in the eggshell integrity of those eggs, as well as a reduced hatching rate.

The experiments performed by Sheahan et al., are basic, but sufficient for their story. In addition, the results reported will be of interest to both the Drosophila oogenesis community of scientists as well as those interested in the function of drd, which is yet unknown. Although the results included in this study does not elucidate the molecular role of drd in epithelia, the authors provide a better understanding of this gene’s role in Drosophila tissues. To be a more solid study, however, there are important controls that are missing throughout the study. These and other comments are outlined below.

Major comments:
1. The Introduction is missing a more complete explanation of the biology of the somatic follicle cells. The authors cite several helpful and comprehensive reviews, but a brief description of the follicle cells (beyond what is included in the first paragraph) will better provide readers of a broader audience the foundation of the organization and life cycle of the follicle cells. For example, it seems important to mention that the follicle cells form a polarized, mono-layered epithelium that lines the germline, with the apical domain facing the germline. This is relevant because it is at this interface that the vitelline membrane and chorion proteins are secreted and form, and that the follicle cells undergo cell death in stage 14 egg chambers.

We have added a new paragraph in the Introduction with additional information about follicle cells and the progression of the egg chamber through oogenesis (lines 60-70).

2. In the Methods, the authors do not explain some of the scoring determinants and/or assays that were used throughout the study (nor do they explain this in the Results text). See major comments #3 and 4 below. Both analyses need to be better described in the Methods (the reference provided is not available on PubMed or Google Scholar).

See responses to comments 3 and 4 below.

3. Specifically, how were eggs determined to be fertilized or not? One widely used approach to do this is to mate drd mutant and knockdown females with a transgenic line (available from BDSC) expressing don juan fused to GFP (dj-GFP). In this line, the sperm tails are easily visible in the egg under a fluorescent dissecting microscope to confirm that fertilization has occurred.

A more complete description of the staging methodology has been included in the methods and results to describe both how unfertilized eggs were recognized and how fertilized embryos were staged (lines 153-159, 310-315).

4. Similarly, it is not clear how the authors determined when embryos arrested. They made the distinction of categorizing pre-gastrula vs. post-gastrula (although how they did this was not stated), but this is a very broad distinction. The data presented shows that 99% of embryos produced from drd mutant females arrest prior to gastrulation. These data suggest that there is a maternal-effect on embryogenesis; this observation will be of interest to the developmental biology community, although an additional experiment is necessary to better clarify the role of drd prior to zygotic genome activation just prior to gastrulation. It would be of great interest to determine in which cell cycle(s) the embryos arrest. Embryos collected from drd mutant and/or RNAi knockdown females can be collected (0-3h post egg-laying), aged for ~3 hours, fixed, and stained for DNA (microtubule staining is helpful as well) to characterize the stage in which the embryos arrest. A presumed maternal-effect lethality would indicate the importance of drd in the syncytial blastoderm in which zygotic genome activation is not yet activated. If the authors are able to make this conclusion based on results that support this hypothesis, this would greatly improve the impact and interest of this work. This could then bolster the claim made in the Discussion (lines 532-533) regarding the developmental arrest hypothesized to occur in these embryos.

We respectfully disagree with the reviewer about the utility and interpretation of the proposed experiment. While the experiment would give us more detailed information about the stage of embryonic arrest, it would not distinguish between a true maternal effect of drd acting in the early embryo vs an indirect effect in which embryonic arrest is caused by a defective eggshell. Given the finding of Elalayli et al 2008 that defective VM crosslinking can cause early embryonic arrest, there is no reason to assume any additional mechanisms in drd mutants. We have revised the discussion language to make this point more explicitly (lines 612-620). We agree with the reviewer that it would be of interest to developmental biologists to understand why defective VM crosslinking causes early embryos to arrest, but that question is beyond the scope of this paper.

5. It was confusing as to why the authors used the drd1 allele solely and did not use the similarly severe allele drdlwf. It would be helpful confirmation to show that the phenotypes reported are consistent across both severe drd alleles, especially since they also included RNAi data to confirm the specificity of the phenotypes reported. In the Discussion (line 567), the authors stat that the drd1 mutant is phenotypically identical to drdlwf, but these experiments were not shown (and perhaps not performed) for the phentoypes assessed in this study. This claim is overstated if those experiments are not included.

The Discussion has been revised to clarify the comparison of the two drd alleles (lines 683-686).

6. There are many different statistical tests used throughout this study and they are stated in the text and figure legends but are not explained in the Methods. These should be better explained in the Methods section to clarify for readers why they used different statistical tests for different analyses.

This information has been added to the final section of the Methods (lines 271-284).

7. The authors evaluated the timing of drd expression in oogenesis but did not show stages prior to drd expression (i.e., previtellogenic stages prior to stage 10). These data should be included to support the expression data referenced from Flybase. Did they indeed observe that Drd is not observed prior to stage 10B? Also, in order for someone to agree with the stages presented in Figure 1, the authors should include DNA staining merged and in separate channels. The GFP visualization alone is not clear enough to determine staging.

We have replaced figures 1 and S3 with new data that includes DAPI staining and shows the absence of GFP expression before stage 10B. The Results and Discussion sections have been updated as well (lines 356-365, 653-654).

8. In Figure 1, it is difficult to discern the outlines of the egg chambers because only the GFP channel is shown. In addition to my suggestion above to include DNA staining, it would be very helpful if the authors presented the egg chambers as individual channels with callout letters instead of all in the same panel. Adding light microscope images or an outline of the egg chambers would be necessary (especially if DNA staining is not included as suggested).

Figures 1 and S3 have been replaced with new data that includes DAPI staining. The GFP and DAPI channels are split into separate panels as requested.

9. This is also true for Figure S3 in which panel B shows an empty field. This is not meaningful if DNA staining or a light microscope image of what is present in the field of view is not included.

This has been corrected in the new figure S3 (see previous responses).

10. Is Drd present in ALL follicle cell (FCs) populations? For example, is Drd-GFP detected in centripetal cells, stretch cells, and in the epithelial monolayer, or is only present in a subset of FCs? Drd-GFP is present in the monolayer on the surface of the egg chamber, as shown in Figure 1, but the focal plane presented in Figure 1 only shows the surface of the egg chamber. Perhaps stretch cells also contain GFP but it’s difficult to say from the stage 10 egg chambers in Figure 1 what the GFP positive cells at the anterior are. Egg chambers shown as a longitudinal section through the middle of the egg chamber would help to elucidate the subpopulations of follicle cells in which Drd-GFP is present. This would be important to bolster their claims in the Discussion (paragraph starting on line 534).

We have updated the Results and Discussion to be more explicit about what we can and cannot conclude about drd expression in different FC subpopulations based on the images (lines 357-365, 653-654). Most of the images in figures 1 and S3 are maximum intensity projections of Z-stacks, which allows us to visualize most FC subpopulations.

11. None of the Western blots presented in the paper include loading controls. It is very difficult to fully believe the conclusions made, especially regarding the lack of protein detected in laid eggs, if a loading control is not included. I do not doubt that the results presented are real, but if these experiments are not well-controlled by showing that the same amount of protein is loaded per lane, the authors cannot make the conclusions presented for those figures.

See response to reviewer #2, point #6 below

12. Line 358: The authors remark that the decreased signal observed in Figure 4A for eggs laid was not consistent across replicates, but this was very striking. Why did the authors present data that was not representative? The decrease was so striking that I was going to suggest that the authors quantify the data but was surprised that they stated inconsistency of this decrease. This suggested a greater necessity to quantify these data. Quantification could be completed using a digital imager and software, or even simply stating how many replicates of the experiment showed the decrease would be perhaps sufficient.

This paragraph has now been clarified (lines 444-447). We only included multiple times post-oviposition in two of our Western blots. One, as shown in figure 4A, showed a decreased signal with increased time post-oviposition, but the other experiment did not show any such decrease.

13. In the data presented in Figure 5C, it was not clear to me if the egg chambers measured were egg chambers (prior to stage 14) or mature oocytes (stage 14). The authors use “egg chambers” and “oocytes” interchangeably in the text. This needs to be better clarified.

We have clarified in both the Methods and Results that the measurements in Fig 5C were of the oocytes within the stage 9-10A egg chambers (lines 237-240, 476).

14. The authors used two follicle cell drivers for their RNAi knockdown experiments: CY-GAL4 and T155-GAL4. Both drivers are expressed in the follicle cells around stage 10, although perhaps at different levels, resulting in varying phenotypic difference. While the varying expressing has been reported before for those drivers, it would be very helpful if the authors also used a follicle cell driver that expresses earlier in oogenesis, such as traffic jam-GAL4 (tj-GAL4) to assess knockdown phenotype, which might be more robust under those conditions.

While one could always test additional GAL4 drivers to probe the temporal requirements for drd expression in the follicle cells, we disagree with the suggestion that use of tj-GAL4 might give a more robust knockdown phenotype. This is because knockdown of drd expression with CY2-GAL4 *fully* mimics all of the observed mutant phenotypes, including female sterility, embryonic arrest, neutral red permeability, and VM protein solubility in laid eggs.

15. It would be extremely helpful for readers if the authors labeled the graphs in Figure 6 differently so that they’re easier to read. The genotypes are very long and for non-geneticists, this will be letter soup. Adding horizontal lines to the top of the graphs (as in the Western blot shown in Figure 7) would greatly help a reader visualize which genotypes are which. For example, a horizontal bar over the first 4 bars would indicate T155-GAL4 and then under that, 2 shorter horizontal lines above the pairs of bars (1 and 2 and then 3 and 4) can be used to indicate the RNAi lines used. Then in the axes for the x-axis, the authors can add “control” or “drd RNAi knockdown” as appropriate.

We agree that the genotypes listed in the original manuscript would be difficult to follow for non-geneticists. We have simplified the genotype names in Figure 6 as suggested.

Minor comments:
1. It would be interesting to the field if the authors could comment about the formation of other important structures in the mature egg that form during oogenesis and may be impacted by proper deposition/development of the eggshell. Do the micropyles and dorsal appendages form properly? Experimental results showing and quantifying eggshell patterning defects might shed light on the precise defects that are found in eggs produced by drd mutant or knockdown females. In addition, results from these experiments might explain some of the phenotypes addressed earlier, such as the reported reduction of fertilized eggs produced by drd mutant or knockdown females.

We agree that these are interesting questions to answer. While we did not note any obvious morphological abnormalities in the eggs laid by mutants, we did not systematically explore egg morphology. We have added a sentence to the Results noting only that such measurements were not conducted (lines 317-319).

2. The authors should add primary antibody dilutions used for Western blot analysis to the Methods.

The revised manuscript now provides the antibodies used for western blots and their concentrations in both the methods (line 214) and the figure legends (lines 427, 459, 579)

3. The authors should include the camera used for confocal microscopy to the Methods.

Image detection is an integrated part of the A1R confocal microscope—there is not a separate camera.

4. Line 197: There is a grammatical error in this line (eclosion “and” placed on yeast paste).

We have corrected grammatical errors (line 228)

5. It would have been better if the authors had used drd/+ controls for their experiments, so if the authors will be repeating experiments, it is suggested that they include this instead of drd/balancer.

We agree with the reviewer that, all else being equal, it would be better to use controls that lack the balancer chromosome. However, doing so would prevent us from using sibling controls and so would introduce new concerns about genetic background effects.

6. In lines 262-264, the authors describe (albeit very briefly) how the data presented in Table 1 was collected. This information should be in the Methods, not the Results. Also, see my comment above about the need for more precise and informative methods regarding these assays.

This information has been moved from the results to the methods (lines 153-159).

7. In the Figures containing graphs, the authors follow a color scheme for the genotypes shown, but the figure legends do not provide a key for the colors used.

We have modified Figure 6 to make the color scheme clearer and have modified the legends of figures 2, 6, and S2 to provide keys for the colors used (lines 403, 549, 881).

8. In the Figures containing graphs, it would be helpful if the authors include the precise “n” for each bar. The figure legends report a range of the number of samples included, but it would be more precise to include the exact “n” for each.

The legends of figures 2, 5, 6, and S2 have been updated (lines 405, 492, 555, 881). Note that in the process of making this correction, we discovered an error in the original legend of figure 2: the lowest n was 115 eggs, not 135. We apologize for this error.

9. I was curious as to why the authors used life span to determine that progeny from females in which germline clones were induced were of the expected genotype. This seems like a good secondary confirmation, but simply sequencing the progeny would be more precise and a better primary confirmation of genotype. Even if the offspring are heterozygous, double peaks should be apparent by sequencing to confirm inheritance of maternal drd1.

We have added more detail about this experiment (see Reviewer 2 minor comment 3 below). In addition, we have modified the language in the Results regarding the progeny lifespan to state that the short lifespan is consistent with the expected drd homozygous germline phenotype, as opposed to our previous language that the short lifespan confirmed the genotype (lines 351-354).

10. The authors mention the number of eggs or egg chambers loaded per lane for the Western blots presented in their data; this information should be included in the Methods and not just in the associated figure legends.

We specify the amount of eggs or egg chambers loaded per lane for Western blots in the Methods in lines 204-207: “For any gel, the same amount of egg chamber equivalents of each sample was loaded (2-4 egg chamber or eggs/lane, see figure legends for experiment-specific details)”

11. Figure legend 4 (lines 362-365): This sentence read awkwardly and should be clarified.

We have clarified the legend of figure 4 (lines 450-455).

12. Figure 5C: Statistical information is missing from the figure legend.

Statistical information has been added to the legend for figure 5C (lines 491-493)

13. Throughout the description of the drd RNAi knockdown phenotypes, the authors simply refer to the drd knockdown females as “knockdown females”. It would be better if the authors add “drd” before this phrase to be more precise.

In the revised manuscript, we refer to RNAi knockdown flies as “*drd* knockdown females”.

14. Line 419: the authors refer to one combination of driver and RNAi transgene to produce a specific effect, but they don’t state that in the text.

The text has been clarified to specify which genotype showed the difference in fertilization (lines 514-515).

15. Table 2 should be reordered to show the control genotypes/phenotypes at the top and the drd RNAi knockdown genotypes/phenotypes in the 2nd row.

Table 2 has been reordered (lines 523-530)

16. There is a concern in the fly community that UAS-Dcr2 presents phenotypes on its own during oogenesis. It would be helpful if the authors used a control that expressed UAS-Dcr2 under GAL4 control instead of GAL4-only controls.

While we are aware of one report that overexpression of Dcr-2 in the germline causes female sterility (El Maghraby et al, Genetics, 2022), we do not know of any reports that Dcr-2 overexpression in the follicle cells affects oogenesis or eggshell synthesis. Thus it does not seem reasonable to repeat all of our RNAi experiments with the additional suggested control.

17. When describing Figure 6 in the text, the authors state that there is a small, but significant increase in the fraction of eggs from drd knockdown females (lines 441-444), but this was only observed for specific combinations of GAL4 and UAS-RNAi lines. This should be better clarified in the text.

This paragraph has been clarified (lines 540-544).

18. When describing the results of Figure 7, the authors report the number of biological replicates that showed the reported results. It was not clear if the authors were reporting that 3 of 3 replicates of GD3367 and 2 of 2 GD15915 displayed the phenotype (lines 460-461). This should be clarified. Similarly, it would be a bit easier to follow if the authors phrased the data at the end of the same paragraph as 2 out of 4 (instead of 2/4) and 1 out of 3 (instead of 1/3).

This paragraph has been clarified (lines 565-571).

19. In the legend for Figure 8, the authors should state that both drd mutant and control siblings were analyzed in the assay shown (and include those genotypes).

The figure 8 legend was revised to include the relevant genotypes of *drd mutants* and controls used in the experiment (lines 599-600).

20. The authors are inconsistent in some of their uses of hyphenation or not throughout the text and figures. For example, sometimes “pre-gastrulation” or “post-gastrulation” are used, and other times both appear without a hyphen. This is also true for “hypo-osmotic”. It is suggested that the authors remain consistent in these terms.

We have corrected the text so that we are consistent in the use of hyphenation throughout the text and figures.

21. The last sentence of the first paragraph on page 26 (lines 560-562) is confusingly written. Perhaps the authors mean to say that the study of Drd could prove to be useful for identifying additional factors needed for eggshell maturation? This should be clarified.

We have modified this sentence to make it clearer (lines 675-678).

22. The authors should add callout letters to Figure 4.

We now include callout letters in Figure 4.

23. In Figure 4, I was confused why 3h and 6h were so close together for the control lanes at the end of panel A. It seems the gel was cropped too closely and the spacing is not the same as for the mutant lanes.

As suggested, the western gel was cropped too closely in Figure 4A. We have corrected this to more accurately represent the spacing of the mutant lanes.

24. In Figure 6, CY2-GAL4 should be consistently capitalized. In the 5th x-axis label for all panels (A-C), CY2-GAL4 is written as “Cy2-GAL4”.

We have simplified the genotypes in Figure 6 and have corrected “CY2-GAL4” such that it is always capitalized.

25. Line 423: Change to “CY2-GAL4” (instead of CY-GAL4).

We have corrected this grammatical error (line 519).

Reviewer #2: Sheahan et al.

The well-written manuscript presents important insight into the function of Dropdead in Drosophila. Loss of Dropdead is known to cause female sterility, but where it is required for fertility and how it impacts fertility was unknown. Taking advantage of the robust genetic tools of Drosophila, in combination with biochemical assessments, the study uncovers that Dropdead is required in the somatic follicle cells during mid to late oogenesis. There Dropdead is required for the proper crosslinking of the vitelline membrane, a key component of the eggshell. The study is well controlled and most phenotypes and changes are quantitatively assessed. A few minor edits will improve the paper, making it easier for a non-expert to fully appreciate the findings.

Minor issues:
1. More details are needed in some of the methods:
a. Visualization of drd expression – how are the egg chambers mounted?

The Methods have been updated with the revised protocol for visualizing drd expression, including more details about sample mounting (lines 175-182).

b. Composition of 5X SDS-PAGE loading buffer

Added to the methods section (lines 201-202)

c. What primaries are used in the western blots and at what concentrations? I understand this is in the figure legends, but it should also be in the methods.

The revised manuscript now provides the antibodies used for western blots and their concentrations in both the methods (line 214) and the figure legends.

d. In immunostaining – how were the intensity measurements normalized?

The intensity measurements were all performed on images taken from the same experiment in one imaging session with constant acquisition settings, so no normalization was required. The Methods section has been revised to clarify this (lines 241-245).

2. Please add data mentioned but not shown.
a. RT-PCR (line 246)

Data have been added to Figure S1 and the text updated accordingly (lines 296, 869-872).

b. Vm26Ab intensity data should be provided as graphs

The data have been added as a new figure (Fig S4). One missing VM width value was added, leading to a slight change in the p-value reported in the Results (line 471) but no change to the conclusions.

3. The results section on the germline clones needs to be expanded to fully explain the clonal system being used. Many Drosophilists think of germline clones as making mosaic ovaries, but you are using the ovoD system. You need to explain how the system works and why the non-heat shock controls are sterile. It will be important to add some clarity on this method to the discussion as well

We have added more detail about this experiment to the Results (lines 335-354) and modified the first paragraph of the Discussion (lines 618-620)

4. Expansion of drd-GAL4 GFP expression data – it would be very helpful for whole ovarioles to be shown (or more stages) to illustrate when it is not present and when it is. Also it appears that only some of the stretch follicle cells express it – is this true?

See responses to Reviewer 1, major comments 7-10.

5. Add n’s to all graphs that don’t have them (Fig. 2, Fig 6)

The legends to figures 2, 5, 6, and S2 have been updated (lines 405, 492, 555, 881).

6. There are no loading controls for the western blots. Please either provide them or better explain why they are not needed.

Reviewer #1 raised a similar concern. We address the concerns of both reviewers below.

To control for protein loading in all Western blot experiments, an equal number of egg chambers or laid eggs were collected from *drd* mutants and sibling controls when preparing samples for analysis. We have clarified the language in the Methods Western blot analysis section (lines 204-208).

While we understand the reviewers’ point that a loading control could be helpful to demonstrate equivalent protein was loaded per lane, particularly in the context of lack of soluble protein in laid eggs, we emphasize that our analysis of the Western blots was qualitative (all or none) and not quantitative. To elaborate, in cases in which there was no protein detected in a lane, these results reflected either: 1) proper eggshell assembly (rendering vitelline membrane and chorion proteins undetectable in laid eggs) or 2) a developmental time point at which a protein had not yet been synthesized (e.g., Cp36 in stage 10 egg chambers). Notably, the expression patterns we report for Cp36 and Vm26Ab in controls are consistent with previous reports (Pascucci et al. 1996, Dev Bio, doi:10.1006/dbio.1996.0188; Manogaran et al. 2004 Dev Bio, doi:10.1016/j.ydbio.2004.02.009). Most importantly, our investigation of *drd* mutant egg chambers revealed a defect in cross-linking of VM proteins, manifested as atypical solubility and detection of VM proteins in Western Blots. Thus, including an additional loading control would not add additional information to support our conclusions.

7. Figure 3 legend text does not state what antibody is used.

We have clarified which antibody was used in the Figure 3 legend (line 427):

We have also made parallel changes to Figure 4 and 7 for consistency (lines 459, 579)

8. Line 347: As expected,…. – it is unclear what sample(s) this sentence is referring to.

We’ve clarified that this sentence is referring to heterozygous controls (line 435).

9. The order of the data presented in Figure 6 does not match the order discussed in the results.

The paragraph has been rewritten to match the figure (lines 534-546).

10. Figure 6 title does not cover everything presented in the figure.

The title of Figure 6 (line 548), as well as Figure 2 (line 399), has been expanded to more accurately reflect the data presented.

11. Figure 8 has very small sample sizes. It would be more convincing with larger numbers.

While the sample sizes in figure 8 are small, the data represent repeated independent trials that gave consistent results. We have modified the Results section to include the number of trials for each condition (lines 588-614).

12. Lines 483-484 – missing figure panel callout or data not shown?

We have added the figure panel callout (line 593).

13. Typos:
a. Line 197: eclosion on placed on yeast paste with sibling males for two days
b. Line 239: The mutation in the latter of these alleles not been molecularly

We have corrected both of these typos such that they now read:

a. Line 228: “…eclosion and placed on yeast paste with sibling males for two days.”

b. Line 288: “The mutation in the latter of these alleles has not been molecularly characterized...”

Reviewer #3: This is a well-written manuscript that investigates the physiological defects that underlie the female sterile phenotype of mutations in the dropdead (drd) gene of Drosophila melanogaster. The authors show that the vitelline membrane portion of the eggshell for eggs laid by drd mutant females, is dye-permeable after the eggs are laid. Normally, the vitelline membrane becomes dye impermeable upon egg activation, when proteins are cross-linked by a poorly understood process. Because the drd sequence suggests that it is an integral membrane protein, consistent with published localization data, the authors hypothesized that Drd protein acts on proteins of the vitelline membrane in a necessary step towards cross-linking. Selective permeability of the vitelline membrane is critical for successful embryogenesis, so that gas exchange can occur, but desication does not.
The authors use flp-FRT-mediated mosaic analysis to show that the critical cell type for Drd gene expression is the somatic follicle cells that produce the components of the eggshell. This result is supported by similar results from dsRNA expression in the follicle cells using in vivo RNAi with follicle cell-specific Gal4 drivers. They further showed that an outer chorion eggshell protein was cross-linked normally in eggs laid by Drd mutant females, indicating a specific defect in vitelline membrane maturation.
To assess whether Drd was directly required during peroxidase-mediated glycosylation, the authors used an exogenous peroxidase method that should render dissected stage 14 immature eggs impermeable to chlorine bleach, even though they have not yet undergone egg activation. Although the control stage 14 immature eggs became impervious to bleach treatment, the immature eggs from stage 14 egg chambers did not.
Based on these results, it appears that Drd is necessary to make vitelline proteins available as substrates for the cross-linking reaction. This is an interesting finding, that will be useful to understanding this complex and poorly understood mechanism to mature the vitelline membrane.

Comments:

For the most part, the experiments are well-chosen and well-described. Sample sizes are appropriate; appropriate controls are used; and the results are critically interpreted.

1. However, the Western blot experiments lack information about replication. The methods are well-described, but it is not clear whether technical and/or biological replicates were performed for each stage examined.

We have added information about biological and technical replicates to the Results text (lines 421, 443, 467, 565-571).

I did not notice any typos, but did not search for them.
