# Supplementary material for: The Drosophila drop-dead gene is required for eggshell integrity
Source: PLoS One. 2023 Dec 5;18(12):e0295412. doi: 10.1371/journal.pone.0295412 (PMC10697589; doi:10.1371/journal.pone.0295412)
Supplement: Attachment — Submitted filename: PLoS One rev2 response to reviewers.docx [file pone.0295412.s010.docx]

**PONE-D-23-14466R2**

**Sheahan et al.**

**The *Drosophila drop-dead* gene is required for eggshell integrity**

**Response to reviewers**

We thank the reviewers for their close reading of our revised manuscript and their comments. Following are the reviewers’ comments with our responses in blue text.

Reviewer #1: The authors addressed most of the textual changes suggested by the reviewers and the manuscript is stronger because of these changes. However, there are important experimental controls that are still missing because the authors did not adequately address the previous reviews. These controls are essential to support the conclusions made and should be included to uphold scientific expectations of well-controlled experimentation.

Major comments:
1. In their response to reviewers and in the manuscript, the authors reference Elalayli et al. (2008) as an example of a study characterizing the mutant phenotype of a vitelline membrane (VM) gene (palisade) with aberrant VM structure. Elalayli et al. perform additional experiments to characterize the mutant phenotype; these experiments were not included in the study by Sheahan et al. despite reviewer requests for similar experiments. For example, confirmation of fertilization using a tagged sperm tail line and evaluation of the stages at which embryonic arrest occurs were shown by Elalayli et al. and suggested by the reviewers, but not included in the revised manuscript. In the revised manuscript, Sheahan et al. use yolk composition to determine fertilization status. I am inclined to accept this less precise method but believe that the lack of staging for the observed arrest is problematic, since the authors’ main conclusions impinge on the timing at which Drd functions in VM formation.
a. Mutant alleles of genes involved in VM composition, secretion, and cross-linking typically lay unfertilized eggs due to defects in eggshell morphology or integrity (resulting in gas exchange and micropyle defects) or cell-cell signaling required for proper dorsal-ventral patterning. Some eggs derived from mutant females, however, are fertilized and those embryos typically arrest in gastrulation (again, likely due to patterning defects). drd-derived embryos may have distinct phenotypes from typical VM gene alleles, but these differences should be better characterized and comparisons should be made to place Drd in specific roles in VM cross-linking, as proposed by the authors.
b. The authors characterized the observed sterility in mutant- and RNAi-derived embryos (Tables 1 and 2) but should perform more precise staging of these embryos to clarify if their arrest is due to processes distinct from VM-associated defects shown for other VM genes. The authors show that 99% of drd¬¬-derived embryos arrest prior to gastrulation, suggesting maternal and not zygotic effects. To my knowledge, syncytial blastoderm arrest (i.e., prior to cellularization) is not usually associated with mutants for genes involved in VM cross-linking. Evidence that cellularization does not occur in drd-derived embryos may suggest another role for Drd in embryogenesis, thereby highlighting the importance of this assay. The results could simply be grouped by pre-cellularization vs. post-cellularization (instead of exact cell cycle stages) among pre-gastrulation arrested embryos. If the authors already have pictures of DAPI-stained embryos, fine-tuned staging quantitation should be straight-forward to perform.
c. Have the authors considered that Drd has two functions – one in VM cross-linking (although the authors suggest that Drd’s effect on VM is likely indirect) and the other in early embryonic signaling/cell cycles (which may be direct or indirect)? If Sheahan et al. quantified the arrest timing of drd1-derived embryos, these results would advance their understanding of Drd’s functions in vivo.

The reviewer states that our use of morphology to score our embryo’s fertilization status and stage of arrest is unacceptably imprecise. While we acknowledge that our methodology is less precise than the methods suggested by the reviewer, we disagree with the reviewer’s contention that more precise methods are required to support the conclusions of the paper. In their comments, the reviewer repeatedly raises the possibility that drd may have two independent functions: one related to VM cross-linking and the other in the early embryo. This possibility appears to be based on the reviewer’s statement that, unlike drd, all other VM mutants result either in unfertilized eggs or in eggs that are fertilized and arrest during or after gastrulation.

We disagree with the reviewer’s contention that all other VM mutants are either fertilized or arrest during gastrulation. As we state in our Discussion, psd mutants, which have a defect in VM cross-linking, are fertilized and arrest pre-gastrulation (Elalayli, 2008). Similarly, class I ndl mutants, which also have a defect in VM cross-linking, are similarly fertilized and arrest pre-gastrulation (Hong and Hashimoto, 1996, Genetics, 143: 1653-1661). It is therefore established in the literature that disruption of VM cross-linking, when not severe enough to cause collapse of the eggs, can result in eggs that are fertilized and arrest during early embryogenesis. Thus, our finding of early embryonic arrest is not specific to drd, and we disagree with the reviewer’s statement that “drd-derived embryos may have distinct phenotypes from typical VM gene alleles”. While we agree that it would interesting to know why such arrest occurs, we believe that answering that question is beyond the scope of this paper.

We have added the Hong and Hashimoto reference to our Discussion (lines 640-643).

2. As pointed out by two reviewers, none of the Western blots presented in this study include loading controls. Although the authors responded as to why they thought this information would not be helpful, I disagree. The lack of protein detected cannot be claimed to be informative if it is not shown experimentally that protein was indeed loaded in those lanes. In the revised manuscript, the authors simply state that the same number of egg chambers or embryos were loaded per lane, but without experimental confirmation, the interpretation of their results, which is vital to the main conclusions of this study, is weakly supported experimentally. Identically loaded Coomassie-stained gels or Ponceau S-stained membranes before blotting would serve as useful loading controls (included in the supplement) if the authors were not able to use a typical loading control for technical reasons.

While we agree that loading controls are essential for quantitative interpretation of Western blots, we argue that such controls are not required for our experiments, nor are they the norm for this type of study. To determine whether loading controls are the norm for Western blots against eggshell proteins, we studied all of the papers listed in Flybase as referencing the VM genes Vm26Aa and Vm26Ab, as well as those papers that cited Pascucci et al., 1996, where the antibodies against these proteins were first reported. We found a total of nine papers, from six different labs, in which Western blots were used non-quantitatively to show the absence or presence of VM cross-linking in laid eggs (1-9). Seven of nine papers did not contain any loading controls, while two papers from one lab (6,7) did report using Ponceau S as a loading control. We further contend that measuring total protein loaded on the gel (such as with Ponceau S) or performing a Western against a cytosolic protein such as actin or beta-tubulin, would not provide any evidence of the solubilization and loading of eggshell proteins. Finally, we reiterate our point from the previous revision that the absence of a Western signal in our control eggs is consistent with all of the previous studies cited here; it is the presence of a signal in our mutant and knockdown eggs that provides the evidence of a role for drd in eggshell crosslinking.

1. Elalayli M, Hall JD, Fakhouri M, Neiswender H, Ellison TT, Han Z, et al. Palisade is required in the Drosophila ovary for assembly and function of the protective vitelline membrane. Dev Biol. 2008;319: 359–369. doi:10.1016/j.ydbio.2008.04.035

2. Ventura G, Furriols M, Martín N, Barbosa V, Casanova J. closca, a new gene required for both Torso RTK activation and vitelline membrane integrity. Germline proteins contribute to Drosophila eggshell composition. Dev Biol. 2010;344: 224–232. doi:10.1016/j.ydbio.2010.05.002

3. Jiménez G, González-Reyes A, Casanova J. Cell surface proteins Nasrat and Polehole stabilize the Torso-like extracellular determinant in Drosophila oogenesis. Gene Dev. 2002;16: 913–918. doi:10.1101/gad.223902

4. Pascucci T, Perrino J, Mahowald AP, Waring GL. Eggshell assembly in Drosophila: processing and localization of vitelline membrane and chorion proteins. Dev Biol. 1996;177: 590–598. doi:10.1006/dbio.1996.0188

5. Manogaran A, Waring GL. The N-terminal prodomain of sV23 is essential for the assembly of a functional vitelline membrane network in Drosophila. Dev Biol. 2004;270: 261–71. doi:10.1016/j.ydbio.2004.02.009

6. Andrenacci D, Cernilogar FM, Taddel C, Rotoli D, Cavaliere V, Graziani F, et al. Specific domains drive VM32E protein distribution and integration in Drosophila eggshell layers. Journal of Cell Science. 2001;114: 2819–29. Available: http://eutils.ncbi.nlm.nih.gov/entrez/eutils/elink.fcgi?dbfrom=pubmed&;id=11683415&retmode=ref&cmd=prlinks

7. Cernilogar FM, Fabbri F, Andrenacci D, Taddei C, Gargiulo G. Drosophila vitelline membrane cross-linking requires the fs(1)Nasrat, fs(1)polehole and chorion genes activities. Dev Genes Evol. 2001;211: 573–580. doi:10.1007/s00427-001-0192-1

8. Heifetz Y, Yu J, Wolfner MF. Ovulation triggers activation of Drosophila oocytes. Dev Biol. 2001;234: 416–424. doi:10.1006/dbio.2001.0246

9. LeMosy EK, Hashimoto C. The Nudel protease of Drosophila is required for eggshell biogenesis in addition to embryonic patterning. Dev Biol. 2000;217: 352–361. doi:10.1006/dbio.1999.9562

3. In the revised manuscript, the authors include a more comprehensive explanation of the ovoD system used to assess the role of drd in the germline vs. somatic cells. This addition is very helpful for the readers. The authors then conclude that drd is only required in somatic cells because 15/17 females were fertile after heat shock treatment. How did the authors assess fertility for this experiment? This information should be added to the Methods. In addition, these results seem contradictory to other results shown in the study wherein larval hatching is severely disrupted (Tables 1 and 2). If the authors used egg-hatching to confirm fertility, then these data do not support their claim that drd-derived embryos exhibit maternal-effect lethality. A better explanation of these contradictory results is requested.

In the ovoD experiment, fertility was assessed by the presence of live larvae. This information has been added to the Methods (line 177). We disagree with the reviewer that the results of this experiment are inconsistent with our other data. This experiment shows that females that are homozygous for a drd mutant in their germline are fertile. We conclude that expression of drd in the female germline is not necessary for fertility. Our other experiments show that drd is expressed in somatic follicle cells and not the germline (figure 1) and that knockdown of drd specifically in the somatic follicle cells recapitulates the drd mutant phenotypes of sterility/early embryonic arrest (table 2), neutral red permeability (figure 6) and defective VM cross-linking (figure 7). Taken together, our data consistently indicate a functional role for drd only in the somatic follicle cells and not in the germline. To clarify this point, we have revised the first paragraph of the Discussion (lines 611-617).

Minor comments:
1. As mentioned previously, UAS-Dcr2 presents phenotypes on its own during oogenesis. The authors would not need to redo every experiment to include this control, as they suggest. Instead, simply collecting and analyzing UAS-Dcr2/+; GAL4/+ (for example) embryos under the same conditions used in the RNAi experiments could be done and compared to the RNAi knockdown results.

We have added new experimental data measuring egg hatching rates in eggs laid by females in which Dcr2 is overexpressed in the follicle cells. With both drivers tested, hatching rates exceeded 98%, indicating that overexpression of Dcr2 does not significantly impact oogenesis. See Methods lines 159-166 and Results lines 515-520.

2. In the revised text, the authors refer to Figure S1 stating that RT-PCR was used to confirm that a shortened drd transcript was produced in drd1 mutants (lines 291-292), but it is not entirely clear how RT-PCR data was included in Fig. S1. I think the authors simply mean that RT-PCR was used to synthesize cDNA that was used for Sanger sequencing, but this is not clear from the text. Either RT-PCR results should be shown (as bands or in a normalized graph), or the Results and Methods should be clarified since the drd1 allele is used throughout their study.

We apologize for the confusion: our use of “RT-PCR” was an abbreviation of “reverse-transcriptase PCR”, not “real-time PCR”. We have clarified this in the Results (line 304). Figure S1 remains as it was in the previous revision, with the cDNA sequence shown in S1b confirming the predicted aberrant splicing from the point mutation in intron 8.

Reviewer #2: The majority of the issues have been addressed. However, I believe the authors' did not upload the revised SFig 1. with the RT-PCR data. Further, the methods are still unclear for how intensity measurements were made (details/program/normalization or not needed). Finally, while the n's are added to Fig 2 and 6 legends, to be consistent with other figures they should be shown in the actual figures.

a. See response to minor comment #2 above.

b. The Methods have been clarified (lines 238-250).

c. We are reluctant to add n-values to figures 2 and 6, as that will make the figures extremely crowded. Keeping the n-values in the figure legends is consistent with figure S2A, which is the figure most similar to figures 2 and 6. The only figure with n-values shown in figure 8, which is a much simpler figure, making the numbers more legible and the figure not overly crowded. Furthermore, having the n-values in figure 8 allows for easier interpretation of the data due to differences in the sample sizes; this is less of a concern for figures 2 and 6.
