# Supplementary material for: The Drosophila drop-dead gene is required for eggshell integrity
Source: PLoS One. 2023 Dec 5;18(12):e0295412. doi: 10.1371/journal.pone.0295412 (PMC10697589; doi:10.1371/journal.pone.0295412)
Supplement: Attachment — Submitted filename: Sheahan et al PLoS One rev3 response to reviewers.docx [file pone.0295412.s011.docx]

**Sheahan et al., response to reviewers**

We thank the reviewers for their close reading of the manuscript and thoughtful comments. Our response to each comment is shown below in blue.

There is a slight difference in the way the Reviewers and I have been looking at the Western images included in the manuscript from the way you want us to look at them. I think we get that the amounts of proteins loaded in the lanes is about equal as you controlled for the starting material that went into the protein preparation protocol.

We also understand that you are not making a quantitative argument; you are using the Western results to highlight drd's function in egg shell assembly/VM protein cross-linking, a process, that is temporally controlled.

We also see that your results for both Cp36 and Vm26Ab are consistent with previous reports.

Our focus now is on the lanes in all the blots where there is no signal. What we are looking for is evidence, that in these lanes, protein was loaded. This is important, because the negative results in the lanes in question are helping you build your story. While doing so, it is important to be convinced that the lanes in question actually received protein extract. This is standard practice these days and a valid request from the Reviewers.

The most straightforward solution would be for you to re-probe the blots shown in the manuscript with anti-lamin, anti-actin, or anti-tubulin (or some other oogenesis-related) antibody to show that the lanes without the signal contain protein. This will help win over the most skeptic of us about the validity of your results. I understand that this is a small point in the larger context of your manuscript, but I believe that not having this control will leave open the discussion whether protein sample was/was not loaded in the lanes without signal.

An additional solution would be for you to review your existing trial and final films from overexposed blots in which the background signal in the lanes without the signal matches the background signal in the lanes with signal. It would be best however to include images of such films (or ones from re-probed blots) in the supplement to explicitly make this point.

In doing so, please organize the data from the additional films into a supplemental figure where the relationship of the newly presented films (and lanes) with that of the ones already included in the manuscript is clearly stated to specifically address this point.

We have created a new supplemental figure, S4_fig, that contains overexposed Western blots in support of figures 4 and 7. In each of these blots, a protein signal is present in some or all the lanes containing laid eggs from control females (the otherwise “blank” lanes). The new figure is referenced in the Results lines 339-340 and 562-3. We hope that this figure is sufficient response to your concerns regarding the interpretation of our Western blot data.

In addition, in response to another concern raised by the reviewers, we have added a sentence to the Discussion (lines 643-645) stating the limitations of our data regarding a possible role for *drd* in the early embryo.
